# The Association Between Magnesium Depletion Score and Depression Among Men in Central Sudan: A Community‐Based Cross‐Sectional Study

**DOI:** 10.1002/hsr2.73448

**Published:** 2026-10-27

**Authors:** Saeed M. Omar, Hatim Y. Alharbi, Afnan A. Alwabili, Eman A. Alotaibi, Sami S. Alharthi, Ishag Adam

**Affiliations:** ^1^ Faculty of Medicine and Health Sciences University of Gadarif Gadarif Sudan; ^2^ Department of Psychiatry College of Medicine Qassim University Buraydah Qassim Saudi Arabia; ^3^ Department of Family and Community Medicine College of Medicine Qassim University Buraydah Saudi Arabia; ^4^ Department of Medicine College of Medicine Taif University Saudi Arabia; ^5^ Department of Obstetrics and Gynecology College of Medicine Qassim University Buraydah Qassim Saudi Arabia

**Keywords:** age, depression, magnesium, men, Patient Health Questionnaire‐9

## Abstract

**Background and Aim:**

Depression is a global public health concern with significant morbidity and mortality. There are a few published reports on the association between the magnesium depletion score (MDS) and depression. In this study, we aimed to assess the level of association between MDS and depression among men in central Sudan.

**Methods:**

A multistage community‐based cross‐sectional survey included adult men in central Sudan from April to June 2025. A face‐to‐face interview was used to collect sociodemographic data and medical history. The Patient Health Questionnaire‐9 (PHQ‐9) was used to assess depression among men. The MDS was computed. Binary multivariable and modified Poisson regression analyses were performed.

**Results:**

Three hundred and twelve men were enrolled. Their median (IQR) age was 50.0 (34.0–64.0) years. Multivariable Poisson analysis showed a positive association between the MDS and the PHQ‐9 score when entering the MDS as either a continuous variable (adjusted relative risk [ARR] = 1.23, 95% confidence interval [CI] = 1.15–1.31) or a categorical variable (the ARR for medium and high MDS were 1.41 (95% CI = 1.23–1.61) and 2.09 (95% CI = 1.60–2.73), respectively). Multivariable binary regression showed a positive association between MDS (continuous) and depression, with an adjusted odds ratio (AOR) of 2.80 (95% CI = 1.35–3.00) and depression. Likewise, medium (AOR = 2.80, 95% CI = 1.28–6.10) and high (AOR = 16.71, 95% CI = 3.91–71.33) MDS were associated with depression.

**Conclusion:**

This study showed a positive association between MDS and depression, indicating a need to further assess the possibility of MDS in depression.

## 1 Introduction

Depression is a highly prevalent mental health disorder affecting 279.6 million people globally in 2019 [1]. It has been estimated that in 2019, there were 4540.4 cases per 100,000 people with depressive disorders in sub‐Saharan Africa [1]. The burden of depression extends beyond individual suffering, as this disorder impacts families, communities, and national economies, with especially notable effects in sub‐Saharan Africa (SSA) [2, 3]. The etiology of depression is complex and multifactorial, involving genetic predispositions, psychosocial stressors, neurobiological imbalances, and lifestyle factors [4, 5]. Several factors such as age [6, 7], marital status [6], level of education [6], alcohol and smoking [8], body mass index (BMI) [6], diabetes mellitus, thyroid disease, and hypertension [6, 8] are associated with depression among adults.

While pharmacological and psychological interventions are the cornerstones of treatment, interest is growing in the role of nutritional factors, particularly micronutrients, healthy dietary patterns, and their association with depression [9]. Magnesium, a trace element, plays an essential role in human physiology, health, and the prevention of disorders and diseases [10], including depression, migraine, and Alzheimer's disease [11]. In the case of depression, serum levels of magnesium [12] and dietary intake of magnesium [13] have been associated with this disorder. However, the human body's magnesium content is difficult to assess and cannot be accurately assessed through traditional methods, such as serum magnesium levels and dietary magnesium intake [14, 15].

Recently, the magnesium depletion score (MDS) has been shown to be a more useful indicator of magnesium status than previously used serum or urine magnesium measurements [16]. Therefore, the MDS is now viewed as a promising tool/score for identifying those patients/individuals with high MDS who may suffer from magnesium deficiency and may benefit from increased magnesium intake to alleviate the risks of magnesium deficiency‐related health conditions, as well as poor sleep quality, systemic inflammation, and cardiovascular disease [6, 16, 17]. This new biomarker/tool assesses magnesium deficiency using four components: “alcohol consumption, current drug use (diuretics and proton pump inhibitor [PPI]), and estimated glomerular filtration rate (eGFR)”. Each of these factors is assigned points, and a higher total score indicates more severe magnesium depletion [16].

In the case of depression, few published data are available regarding an association between the MDS and this disorder, and most consist of USA national survey data [6, 18, 19]. Therefore, similar research is needed for a broader range of populations, especially those in SSA countries, which are burdened by poor mental health and nutrition [3]. For example, in Ethiopia, the prevalence of depression among adults was 18.0% [8]. SSA countries also face substantial malnutrition, including low intake of micronutrients. In different regions of Sudan, including the eastern area, low intake of micronutrients has been accompanied by a rising prevalence of depression among various population groups, including adolescents and adults [7, 20]. This has been further exacerbated by ongoing armed conflicts, with 62% of internally displaced persons now classified as depressed [7].

In Central Sudan, as in many regions in SSA, people face great health challenges in the presence/form of ongoing armed conflict, including a high burden of mental health disorders and potential nutritional deficiencies [21]. However, specific research examining the association between MDS and depression in this particular population is scarce. Understanding these associations is essential for developing targeted public health interventions and improving mental health outcomes. The present study aimed to assess the association between MDS and depression among adult men in central Sudan. We included only men in this study because, according to Sudanese tradition, alcohol consumption (one component measured to determine the MDS) is not considered a female habit.

## 2 Methods

### 2.1 Study Design and Setting

A community‐based cross‐sectional survey was conducted at Elrikieb, which is a village in the eastern part of Gezira, in Central Sudan, from April to June 2025. Gezira State is located in central Sudan between the two largest rivers in Sudan (the Blue and White Nile Rivers). In Gezira State, there is one of the largest irrigated agricultural schemes in the world. As previously stated [22], Elrikieb is one of the biggest villages in the eastern Gezira area and was selected because it comprises four sub‐villages, and its inhabitants could resemble those of the entire Gezira State. In the present study, the authors strictly adhered to the “Strengthening the Reporting of Observational Studies in Epidemiology (STROBE) guidelines” [23] (Supporting File).

### 2.2 Study Population and Sampling

We have previously reported the association between proton pump inhibitors and depression in both females and males in the same community [22]. In the current study, the population consisted of adult men aged 18 years or older residing in the selected communities. This study included only adult men because alcohol consumption, one of the components used to determine the MDS, is not common among Sudanese women, unlike in other African and Western countries. The exclusion criteria were as follows: men unable to provide informed consent, as well as men with severe cognitive impairment; hence, they were unable to complete the Patient Health Questionnaire‐9 (PHQ‐9).

In this survey, a multistage stratified random sampling approach (lottery) was employed to select the study participants (men), with households randomly selected from each of the four sub‐villages. The number of selected households was proportional to the number of households in each sub‐village in Elrikieb. From each eligible household, one adult male was randomly selected using a lottery method. When there was no male in the selected household, or they refused to participate, the next household was selected.

### 2.3 Sample Size Calculation

A sample size of 312 men was calculated based on the assumption that 30.0% of men would have depression. This assumption was based on previous findings from Sudan, which showed that 44% of the older people had depression [24]. We assumed that 48.0% of the depressed men would have a medium or high MDS, while 25.0% of the men without depression would have a medium or high MDS. This sample size would have 80% power and an alpha level of 0.05.

### 2.4 Data Collection

After the adult men signed informed consent forms, data were collected by trained male medical officers (medical graduates) via face‐to‐face interviews using a questionnaire in the local Arabic language. The questionnaires collected data on sociodemographic factors, such as age (years), education level (equal to or less than or more than the secondary level, which is equal to 8 years of education), marital status, employment status, smoking status, medications used (particularly diuretics and PPIs), and medical history, including diabetes mellitus, thyroid disease, and hypertension, in private. Each man's weight and height were measured using standard procedures, and their body mass index (BMI) was calculated as weight (kg) divided by the square of height (m).

Hypertension was considered if “there was a previous diagnosis of hypertension and use of medications, or it was considered newly diagnosed hypertension, determined if the systolic blood pressure was greater than or equal to 140 mmHg, the diastolic blood pressure was greater than or equal to 90 mmHg, or both. Men were considered to have diabetes mellitus if they had already been diagnosed with it”.

### 2.5 Assessment of Depression

As in our previous report [22], the Arabic version of the PHQ‐9 questionnaire was used to screen men for depression. The PHQ‐9 is a brief, widely validated tool available in several languages for screening, as well as monitoring the severity of depression. The Arabic‐validated version of the PHQ‐9 was used before in Sudan [25] It consists of 9 items, each scored from 0 to 3, yielding a total score ranging from 0 to 27 [26]. In this survey, we adopted a score of ≥ 8 as the cut‐off for clinically significant depression [27], and this was based on previous studies that showed excellent accuracy [28, 29]. Men with a score of ≥ 8 were considered to have significant clinical depression and were referred for further evaluation and management.

### 2.6 Assessment of the Magnesium Depletion Score

As previously stated, a composite MDS was calculated based on the following four indicators, each contributing 1 point if present [16]:

− One point was given for alcohol abuse/consumption.
− Diuretic Use: Self‐reported current use of any diuretic medication, such as furosemide or hydrochlorothiazide, as prescribed by a healthcare professional. One point was given for current use.
− Proton Pump Inhibitor Use: Self‐reported current use of any PPI medication, such as omeprazole or pantoprazole, as prescribed by a healthcare professional. One point was given for current use.
− Kidney function: An eGFR < 90 mL/(min·1.73 m^2^) was assigned 1 point. An eGFR < 60 mL/(min·1.73 m^2^) was given 2 points.

The eGFR was calculated based on the serum creatinine level. “The Joint National Kidney Foundation and the American Society of Nephrology Task Force recommend using the Chronic Kidney Disease Epidemiology Collaboration (CKD‐EPI) Creatinine Equation (2021) to calculate eGFR [30, 31] ”. The 2021 CKD‐EPI eGFRcr equation estimates glomerular filtration rate (GFR) using age and serum creatinine, as expressed below, as a single equation [32, 33].

Then, when computing continuous MDS scores, we divided them into three categories: Low (0 points), Medium (1–2 points), and High (3 or more points). The low score (0) was considered the reference one.

### 2.7 Data Analysis

All statistical analyses were performed using SPSS for Windows (version 27.0). The Shapiro–Wilk test indicated that age, BMI, PHQ‐9 score, and MDS were not normally distributed. Hence, their values were expressed as medians (interquartile range [IQR]). A modified Poisson and binary logistic regression analysis (univariable and multivariable) was performed, with the PHQ‐9/depression score (continuous and classified as yes/no) as the dependent variable. The sociodemographic characteristics (men's age, education level, occupation, and family history of mental illness) and MDS were the independent variables. MDS was entered as a continuous variable and then categorized (MDS = 0, MDS = 1, MDS = 2, or MDS ≥ 3), and depression was also included. Variables with *p*‐values < 0.2 in the univariable analyses (Poisson and binary) were included in the multivariable analysis (enter method), adjusted for potential confounders [34]. There was no multicollinearity (variance inflation factor < 4). Adjusted odds ratios (AORs), 95% confidence intervals (CIs), and correlation coefficients were reported. A two‐sided *p*‐value < 0.05 was considered statistically significant.

## 3 Results

### 3.1 General Characteristics

Three hundred and twelve men were enrolled in the study. Their median (IQR) age was 50.0 (34.0–64.0) years, and their median BMI was 21.4 (18.9–24.5) kg/m^2^. Overall, 82/312 (26.3%) men had an education level at or above secondary level, and 59/312 (18.9%) were unemployed. In total, 244/312 (78.2%) men were married, 143/312 (45.8%) were smokers, and 71/312 (22.8%) were alcoholics. Healthwise, 110/312 (35.3%) men were hypertensive, and 39 (12.5%) men had diabetes mellitus. Of the 39 men with diabetes mellitus, 12 were on lifestyle and diet control only, 22 were on oral hypoglycemic drugs, and 5 were on insulin. Regarding medication use, 49/312 (15.7%) men used proton pump inhibitors, and 8/312 (2.6%) used diuretics. Of the 312 men, 172 (55.1%), 124 (39.7%), and 16 (5.1%) had low, medium, and high MDSs, respectively (Table 1).

**Table 1:** General characteristics of men in East Gezira, Sudan, 2025 (*n* = 312).

Characteristics		Median	Interquartile range
Age, years		50.0	34.0–64.0
Body mass index, kg/m^2^		21.4	18.9–24.5
		Number	Percentage
Education status	≥ Secondary	82	26.3
	< Secondary	230	73.7
Occupation status	Employed	13	4.2
	Self‐employed	240	76.9
	Unemployed	59	18.9
Marital status	Married	244	78.2
	Unmarried/divorced	68	21.8
Smoking status	No	169	54.2
	Yes	143	45.8
Alcohol consumption	No	241	77.2
	Yes	71	22.8
Diuretic intake	No	304	97.4
	Yes	8	2.6
Proton pump inhibitor intake	No	263	84.3
	Yes	49	15.7
Diuretic, proton pump inhibitor, or both	No	256	82.1
	Yes	56	17.9
Having diabetes mellitus	No	273	87.5
	Yes	39	12.5
Hypertension	No	202	64.7
	Yes	110	35.3
Magnesium depletion score	Low (0)	172	55.1
	Medium (1–2)	124	39.7
	High (≥ 3)	16	5.1

### 3.2 Factors Associated With the PHQ‐9 Scores

Univariable Poisson regression analysis identified age, education, occupation, marital status, smoking, alcohol consumption, and use of proton pump inhibitors as factors associated with the PHQ‐9 scores. In contrast, diuretic use, hypertension, and diabetes mellitus were not associated. Shifting the variables to a multivariable Poisson regression revealed that age, occupation, marital status, smoking, alcohol consumption, and the use of proton pump inhibitors remained associated with the PHQ‐9 scores. After adjusting for the confounders, entering the MDS as either a continuous variable (ARR = 1.23, 95% CI = 1.15–1.31) or a categorical variable (the ARR for medium and high MDS were 1.41 (95% CI = 1.23–1.61) and 2.09 (95% CI = 1.60–2.73), respectively), it was associated with the PHQ‐9 scores. Entering the components of the MDS (alcohol consumption and proton pump inhibitor use) into each model separately gave ARR values of 1.31 (95% CI = 1.11–1.53) and 1.56 (95% CI = 1.34–1.81), respectively (Table 2).

**Table 2:** Univariate and multivariable modified Poisson analysis of factors associated with depression score among men in East Gezira, Sudan, 2025 (*n* = 312).

		Univariable analysis			Multivariable analysis		
Characteristics		Relative risk	95% CI	*p* value	Adjusted relative risk	95% CI	*p* value
Age, years		0.99	0.991–0.998	0.002	0.995	0.990–0.999	0.031
Body mass index, kg/m^2^		0.99	0.98–1.01	0.093	0.99	0.98–1.01	0.252
Magnesium depletion score*		1.21	1.13–1.28	< 0.001	1.23	1.15–1.31	< 0.001
Education status	≥ Secondary	Reference				Reference	
	< Secondary	1.17	1.02–1.33	0.018	1.33	1.16–1.53	< 0.001
Occupation status	Employed	Reference				Reference	< 0.001
	Self‐employed	1.01	0.69–1.45	0.992	0.89	0.61–1.29	0.549
	Unemployed	1.40	1.18–1.66	< 0.001	1.40	1.17–1.66	< 0.001
Marital status	Married	Reference				Reference	
	Unmarried/divorced	0.77	0.67–0.88	< 0.001	0.74	0.64–0.85	< 0.001
Smoking status	No	Reference				Reference	
	Yes	0.76	0.68–0.86	< 0.001	0.82	0.72–0.93	0.003
Alcohol consumption*	No	Reference			Reference		
	Yes	1.30	1.13–1.48	< 0.001	1.31	1.11–1.53	< 0.001
Diuretic intake	No	Reference					
	Yes	0.94	0.65–1.36	0.743			
Proton pump inhibitor intake*	No	Reference				Reference	
	Yes	1.60	1.39–1.85	< 0.001	1.56	1.34–1.81	< 0.001
Diuretic, proton pump inhibitor, or both*	No	Reference				Reference	
	Yes	1.51	1.31–1.74	< 0.001	1.49	1.29–1.73	< 0.001
Having diabetes mellitus	No	Reference					
	Yes	0.93	0.78–1.11	0.466			
Hypertension	No	Reference					
	Yes	1.06	0.93–1.21	0.321			
Magnesium depletion score*	Low (0)	Reference				Reference	
	Medium (1–2)	1.37	1.21–1.56	< 0.001	1.41	1.23–1.61	< 0.001
	High (≥ 3)	1.64	1.29–2.09	< 0.001	2.09	1.60–2.73	< 0.001

* Entered one by one into the models.

### 3.3 Factors Associated With Depression

Of the 312 participants, 39 (12.5%) had depression. Univariable analysis identified age (OR = 0.97, 95% CI = 0.95–0.99), being married (OR = 2.59, 95% CI = 1.27–5.28), and high MDS (OR = 4.75, 95% CI = 1.45–15.52) as associated with depression, whereas education, employment, BMI, alcohol, smoking, diabetes mellitus, and hypertension were not associated with depression (Table 3). Inclusion of variables with *p* < 0.20 in the multivariable binary regression revealed that MDS (as a continuous variable) had an AOR of 2.80 (95% CI = 1.35–3.00) and was associated with depression. Likewise, men with a medium MDS (AOR = 2.80, 95% CI = 1.28–6.10) or a high MDS (AOR = 16.71, 95% CI = 3.91–71.33) had increased odds of depression. Proton pump inhibitor use was also associated with depression (AOR = 3.36, 95% CI = 1.51–7.47) (Table 4).

**Table 3:** General characteristics and univariable binary analysis of factors associated with depression among men in East Gezira, Sudan, 2025 (*n* = 312).

		Men with depression (*n* = 39)	Men without depression (*n* = 273)	Univariable analysis	
Characteristics		Median (IQR)	Median (IQR)	Odds ratio (95% CI)	*P* value
Age (years)		40.0 (23.7–60.5)	50.0 (36.0–65.0)	0.97 (0.95–0.99)	0.024
Body mass index (kg/m^2^)		20.3 (18.9–23.3)	21.7 (18.9–24.6)	0.97 (0.91–1.04)	0.553
		Number (percentage)	Number (percentage)		
Education status	≥ Secondary	14 (35.9)	68 (24.9)	Reference	
	< Secondary	25 (64.1)	205 (75.1)	0.59 (0.29–1.20)	0.148
Occupation status	Employed	2 (5.1)	11 (4.0)	Reference	
	Self‐employed	32 (82.1)	208 (76.2)	0.84 (0.17–3.99)	0.833
	Unemployed	5 (12.8)	54 (19.8)	0.50 (0.08–2.97)	0.453
Marital status	Married	24 (61.5)	220 (80.6)	Reference	
	Unmarried/ divorced	15 (38.5)	53 (19.4)	2.59 (1.27–5.28)	0.009
Smoking status	No	20 (53.3)	149 (54.6)	Reference	
	Yes	19 (48.7)	124 (45.4)	1.14 (0.58–2.23)	0.699
Alcohol consumption	No	30 (76.9)	211 (77.3)	Reference	
	Yes	9 (23.1)	62 (22.7)	1.02 (0.46–2.26)	0.595
Diuretic intake	No	38 (97.4)	266 (97.4)	Reference	
	Yes	1 (2.6)	7 (2.6)	1.01 (0.12–8.35)	0.999
Proton pump inhibitor intake	No	27 (69.2)	236 (86.4)	Reference	
	Yes	12 (30.8)	37 (13.6)	2.83 (1.32–6.08)	0.007
Diuretic, proton pump inhibitor, or both	No	27 (69.2)	229 (83.9)	Reference	
	Yes	12 (30.8)	44 (16.1)	2.31 (1.09–4.91)	0.029
Having diabetes mellitus	No	35 (89.7)	238 (87.2)	Reference	
	Yes	4 (10.3)	35 (12.8)	0.77 (0.26–2.32)	0.651
Hypertension	No	26 (66.7)	176 (64.5)	Reference	
	Yes	13 (33.3)	97 (35.5)	0.90 (0.44–1.84)	0.788
Magnesium depletion score	Low (0)	15 (38.5)	157 (57.5)	Reference	
	Medium (1–2)	19 (48.7)	105 (38.5)	1.89 (0.92–3.89)	0.089
	High (≥ 3)	5 (12.5)	11 (4.0)	4.75 (1.45–15.52)	0.010

**Table 4:** Multivariable binary analysis of factors associated with depression among men in Gezira State in Sudan, 2025 (*n* = 312).

Variable		Adjusted odds ratio	95% CI	*p* value
Age, years		0.97	0.95–1.01	0.103
Magnesium depletion score*		2.01	1.35–3.00	0.001
Education status	≥ Secondary	Reference		
	< Secondary	0.65	0.29–1.42	0.282
Marital status	Married	Reference		
	Unmarried/ divorced	1.91	0.73–4.93	0.181
Proton pump inhibitor intake*	No	Reference		
	Yes	3.36	1.51–7.47	0.003
Diuretic, proton pump inhibitor, or both*	No	Reference		
	Yes	2.78	1.26–6.14	0.011
Magnesium depletion score category*	Low (0)	Reference		
	Medium (1–2)	2.80	1.28–6.10	0.009
	High (≥ 3)	16.71	3.91–71.33	< 0.001

* Entered one by one into the models

## 4 Discussion

The main findings of the current study were that each 1‐point increase in MDS was associated with a 0.23‐point increase in the depression score (PHQ‐9) in the continuous analysis (ARR = 1.23, 95% CI = 1.15–1.31). Moreover, the odds of depression increased by 12.0% (OR = 1.12, 95% CI = 1.04–1.21) for MDS. The use of proton pump inhibitors was independently associated with an increased likelihood of having depressive symptoms (OR = 1.24, 95% CI = 1.04–1.46; OR = 1.95, 95% CI = 1.64–2.32), in alignment with the results of three surveys from the National Health and Nutrition Examination Survey (NHANES) [6, 18, 19]. In a study examining 18,247 adults (aged 20–60 years) in a US population, Cai et al. reported that MDS as a continuous variable (OR = 1.15, 95% CI = 1.04–1.26) or as a categorized variable (MDS = 1, OR = 1.03; MDS = 2, OR = 1.41; and MDS ≥ 3, OR = 1.58 [1.06, 2.35]) was associated with an increased OR for depression [18]. Likewise, analysis of data from 20,893 NHANES participants by Lu et al. revealed that a 1‐point increase in the MDS resulted in a 0.23‐point increase in PHQ‐9 score and a 12% increase in the odds of depressive symptoms [19]. Zhao and Jin reported a positive correlation between MDS and depression in 13,197 participants, and they observed a 19% increase in depression likelihood in the medium MDS group (OR = 1.19, 95% CI = 1.01–1.41) and a 58% increase in the high group (OR = 1.58, 95% CI = 1.21–2.07) when the low MDS subgroup was used as a reference [6].

Taken together, the prevailing evidence suggests that low magnesium levels can be associated with depression. The use of both diuretics and proton pump inhibitors can reduce body magnesium levels by either reducing intestinal absorption or increasing renal excretion [35, 36, 37]. Impaired renal function, as reflected in the GFR, can also disrupt the magnesium–electrolyte balance [38, 39]. Likewise, alcohol consumption can lead to magnesium depletion due to poor nutritional intake, gastrointestinal malabsorption, and increased urinary excretion [40]. However, the exact mechanism by which low magnesium levels may trigger depressive symptoms remains unclear.

Magnesium has been reported to act as an antagonist of the N‐methyl‐d‐aspartate (NMDA) receptor. NMDA is a modulator of gamma‐aminobutyric acid (GABA) neurotransmission and can influence the synthesis of both serotonin and norepinephrine, both of which are implicated in mood regulation [41, 42, 43]. Thus, magnesium deficiency can contribute to the development and exacerbation of depression [44, 45].

Despite magnesium's physiological importance, its deficiency is a common global issue that often remains underdiagnosed due to challenges in accurate assessment. Serum magnesium levels do not always reflect total body magnesium stores, as only a small fraction is present extracellularly [14, 15]. Because less than 1% of total body magnesium is present in serum, serum magnesium concentration does not truly reflect total body magnesium content or intracellular magnesium content [15]. Therefore, a comprehensive approach, such as the MDS, that incorporates clinical indicators of increased magnesium loss or reduced absorption may provide a more practical and holistic assessment of the magnesium status of human populations in community settings.

### 4.1 Strengths and Limitations

In the current study, we assessed only men, as alcohol consumption is a man's habit in Sudan and a component of the MDS. Excluding women from this study for this reason could have introduced selection bias. Fluctuations in estrogen levels have recently been shown to influence magnesium absorption, distribution, and retention in women. Moreover, dietary magnesium intake and lifestyle factors may differ between men and women [46]. A cross‐sectional study cannot determine the cause‐and‐effect relationship between MDS and depression and will require larger longitudinal studies for confirmation. Information on the quantity of alcohol consumed was not collected. Important variables such as dietary magnesium intake, serum magnesium, socioeconomic status, sleep quality, physical activity, chronic inflammatory conditions, medication indications, conflict‐related trauma or displacement, and previous psychiatric history were not investigated.

## 5 Conclusion

This study showed a positive association between MDS and depression. The findings have clinical implications: use MDS to identify people at risk and promote healthy diets rich in magnesium. Larger studies with different types of people are needed.

## Author Contributions

**Saeed M. Omar:** conceptualization, methodology, formal analysis, writing – original draft, writing – review and editing. **Hatim Y. Alharbi:** conceptualization, investigation, formal analysis. **Afnan A. Alwabili:** writing – original draft, writing – review and editing. **Eman A. Alotaibi:** Investigation, Formal analysis. **Sami S. Alharthi:** writing – review and editing, writing – original draft. **Ishag Adam:** conceptualization, writing – original draft, writing – review and editing, supervision.

## Funding

The authors have nothing to report.

## Ethics Statement

This study was conducted in accordance with the principles outlined in the Declaration of Helsinki and good clinical research practices. Ethical approval was granted by the Research Ethics Committee of the Faculty of Medicine and Health Sciences at the University of Gadarif in Gadarif, eastern Sudan (Ref. #2025, 04, E).

## Consent

Written informed consent was obtained from all participants.

## Conflicts of Interest

The authors declare no conflicts of interest.

## 1 Transparency Statement

Saeed M. Omar affirms that this manuscript is an honest, accurate, and transparent account of the study being reported; that no important aspects of the study have been omitted; and that any discrepancies from the study as planned have been explained.

## Supporting information


Supporting File

## Data Availability

The data of the current study will be available from the corresponding author upon reasonable request.
